# Providing education on evidence-based practice improved knowledge but did not change behaviour: a before and after study

**DOI:** 10.1186/1472-6920-5-40

**Published:** 2005-12-19

**Authors:** Annie McCluskey, Meryl Lovarini

**Affiliations:** 1 School of Exercise and Health Sciences, University of Western Sydney (Campbelltown Campus, Blg 24), Locked Bag 1797, Penrith South DC, New South Wales 1797, Australia

## Abstract

**Background:**

Many health professionals lack the skills to find and appraise published research. This lack of skills and associated knowledge needs to be addressed, and practice habits need to change, for evidence-based practice to occur. The aim of this before and after study was to evaluate the effect of a multifaceted intervention on the knowledge, skills, attitudes and behaviour of allied health professionals.

**Methods:**

114 self-selected occupational therapists were recruited. The intervention included a 2-day workshop combined with outreach support for eight months. Support involved email and telephone contact and a workplace visit. Measures were collected at baseline, post-workshop, and eight months later. The primary outcome was knowledge, measured using the Adapted Fresno Test of Evidence-Based Practice (total score 0 to 156). Secondary outcomes were attitude to evidence-based practice (% reporting improved skills and confidence; % reporting barriers), and behaviour measured using an activity diary (% engaging/not engaging in search and appraisal activities), and assignment completion.

**Results:**

Post-workshop, there were significant gains in knowledge which were maintained at follow-up. The mean difference in the Adapted Fresno Test total score was 20.6 points (95% CI, 15.6 to 25.5). The change from post-workshop to follow-up was small and non-significant (mean difference 1.2 points, 95% CI, -6.0 to 8.5). Fewer participants reported lack of searching and appraisal skills as barriers to evidence-based practice over time (searching = 61%, 53%, 24%; appraisal 60%, 65%, 41%). These differences were statistically significant (p = 0.0001 and 0.010 respectively). Behaviour changed little. Pre-workshop, 6% engaged in critical appraisal increasing to 18% post-workshop and 18% at follow-up. Nearly two thirds (60%) were not reading any research literature at follow-up. Twenty-three participants (20.2%) completed their assignment.

**Conclusion:**

Evidence-based practice skills and knowledge improved markedly with a targetted education intervention and outreach support. However, changes in behaviour were small, based on the frequency of searching and appraisal activities. Allied health educators should focus more on post-workshop skill development, particularly appraisal, and help learners to establish new routines and priorities around evidence-based practice. Learners also need to know that behaviour change of this nature may take months, even years.

## Background

Regardless of the desire to keep up to date, behaviour change is difficult. Even the most motivated of health professionals face barriers when attempting to stay up to date by finding, reading and using research. The primary barriers reported by allied health professionals for not using research in practice are a perceived lack of time, and lack of skills and knowledge when searching for and appraising research [1-5]. Yet little has been done in allied health to address these barriers.

Continuing professional education, particularly workshops can improve knowledge but the impact on behaviour is less impressive. In a recent randomised controlled trial, Taylor and colleagues found that after six months, a half-day workshop on critical appraisal had improved knowledge but not the evidence-seeking behaviour of health professionals, including physiotherapists [6]. These findings are consistent with systematic reviews on the effectiveness of teaching evidence-based medicine and critical appraisal skills [7-9]. Therefore, a major challenge for educators is effecting knowledge transfer and behaviour change.

What appears to help promote behaviour change is partly the way in which education is delivered, and partly the provision of follow-up [10]. In their Cochrane review, Thompson O'Brien and colleagues [10] recommended that researchers investigate components of workshops that contribute to effectiveness, such as practicing skills during and after a workshop, providing follow-up outreach support, and providing feedback on behaviour change. These collective strategies are often referred to as a 'multifaceted' intervention, where 'champions' of evidence-based practice market concepts to the profession, links are maintained with learners after training, and reminders and feedback are used to encourage behaviour change [10-14].

The effectiveness of a multifaceted intervention, aimed at improving evidence-based practice behaviours has received limited attention by allied health researchers. In addition to Taylor and colleagues [6], Stevenson, Lewis and Hay completed another randomised controlled trial involving education and training of British physiotherapists [15]. While their one-day workshop on searching and appraisal skills was interactive, the overall intervention was not multifaceted, and no follow-up support was provided. Furthermore, the authors focussed on measuring (and changing) attitudes using self-report measures. No objective tests of skill or knowledge, nor prospective measures of behaviour were used. Another randomised controlled trial [16] has reported the use of a multi-faceted intervention program, designed to increase the use of evidence-based practice. However, this study was conducted with public health physicians, not allied health professionals, and was published in 2003 after the current study had finished. Therefore, this important study by Forsetlund and colleagues will be discussed later.

In summary, the current study adopted a multi-faceted approach involving an interactive 2-day workshop for occupational therapists, written materials, use of opinion leaders and champions, a discussion list and information service, and outreach follow-up support. At the time of planning this study (2001), skills-based workshops were commonly used to introduce allied health professionals to evidence-based practice, but no efficacy studies had been reported.

The aim of this before and after study was to measure the effect of a multifaceted intervention on evidence-based practice, on the knowledge and skills, behaviour and attitudes of occupational therapists. We hypothesised that post-intervention, participants would demonstrate improved skills and knowledge, report fewer barriers to adopting evidence-based practice, particularly lack of skills and knowledge, use their skills more frequently at work, and that these changes would be maintained over time.

## Methods

The study commenced in November 2001 and concluded in March 2003, with approval from the University of Western Sydney ethics committee. A before and after study design was used. There was no control group. The intervention is described in detail below.

### Recruitment

To be included, participants had to be a qualified, employed occupational therapist, working in the state of New South Wales, Australia during the period of the study. An advertisement was distributed by email and post, to a wide range of private and public employers. Therapists were invited to participate, and to encourage junior and senior colleagues, as well as friends to enroll. This method of 'snowball' sampling aimed to recruit at least 100 occupational therapists. No eligibility restrictions were placed on health sector, work location or clinical specialty. A total of 232 therapists expressed interest, with 114 being recruited.

### Intervention

The primary intervention was a 2-day workshop on evidence-based practice, held in February 2002. Of the 114 recruited, 106 attended the workshop. To accommodate the large number of participants, three 2-day workshops were conducted over a month. Each workshop provided the same content but on different weekends over a month. The authors conducted the workshops in a metropolitan city, with the assistance of a health librarian. The authors were considered 'expert' clinicians, each having over 15 years of occupational therapy experience in public and private health sectors and had attended short skills-based courses on evidence-based practice. This experience and knowledge helped us to choose important occupational therapy questions and studies for appraisal during the workshop.

The workshop included lectures, practical sessions and small group discussion focussed around six topics: the process of evidence-based practice; writing focussed clinical questions; searching electronic databases; critical appraisal of qualitative and quantitative research; interpreting statistics in randomised controlled trials; and overcoming barriers/making the change to evidence-based practice.

The workshop used principles of andragogic or adult learning theory, [17] and social cognitive theory [18] to help participants engage with the new 'innovation', evidence-based practice. Social cognitive theory aims to promote learning and behaviour change by increasing the self-efficacy of learners (see Bradley and colleagues for an excellent review) [19]. The format and content of sessions, and clear learning objectives were developed with a steering committee comprising five occupational therapy clinicians, two allied health experts in evidence-based practice, a health librarian and two service users. During the workshop, new skills and knowledge such as writing clinical questions, searching databases, and interpreting statistics were modelled by presenters, using worked examples. Time was set aside after each session for individuals to reflect, consider how they would apply the new skills and knowledge, and write personal learning goals to be achieved post-workshop. During the final workshop session, the change process and potential barriers to adopting evidence-based practice were actively discussed. A short presentation describing Rogers five-stage model of innovation diffusion [20] and Prochaska and DiClemente's transtheoretical model of change [21,22] provided the stimulus for discussion.

To promote post-workshop skill development participants were invited to develop a learning contract which included a critically appraised topic (CAT). These CATs could be completed individually or in pairs as an optional 'assignment'. Participants wrote a clinical question about the effectiveness of an occupational therapy intervention, to focus their CAT. By writing a CAT, it was hoped that participants would develop improved search and appraisal skills, and document the practice implications of research appraised. Although the use of CATs as a learning assignment had not previously been reported in this context, hypothetical assignments have been used [16].

The follow-up outreach support consisted of regular email and telephone contact, and an optional workplace visit. Support was provided by an expert occupational therapy practitioner (ML), employed as a research assistant/project manager. The visits (n = 82) provided help with searching and appraisal, and monitored progress with the assignment. An email list was set up to facilitate communication. Information distributed via this list included resources and websites and answers to frequently asked questions. Reminders and individual feedback were provided about the assignment. Between March 2002 and February 2003, approximately 180 email messages were sent to this list, most by the project manager (ML). Approximately 225 email messages, mostly questions, were received from participants. The number of phone calls made and received was not logged. Completed CATs were presented at a one-day conference in February 2003, and uploaded to a new website.

### Outcome measures

A written questionnaire and the Adapted Fresno Test of competence in evidence-based medicine [23] were used to measure knowledge, the primary outcome. The questionnaire also captured data on attitudes to evidence-based practice (secondary outcome). A written activity diary and assignment completion were used to measure behaviour (secondary outcome). Copies of the measures are available upon request from the first author (AM).

All measures except the activity diary were collected before, immediately after (i.e. at the end of day two) and 8-months post-workshop. The activity diary was collected prospectively on five occasions: for 3-weeks pre-workshop, then for 8-months during 2002 with bi-monthly collection (March-April; May-June; July-August; September-October).

### Questionnaire

The 8-page questionnaire was developed from existing instruments [3,23-25]. Pilot-testing was conducted with eight occupational therapists from other states and territories in Australia. Minor changes were made to the content, layout and formatting in response to feedback. Participants completed the questionnaire on three occasions. The first questionnaire was mailed out, and returned on the first morning of the workshop (baseline). The second questionnaire was completed at the end of the workshop (post-workshop). The third and final questionnaire was distributed and returned by mail, approximately eight months after the workshop (follow-up).

The questionnaire contained three sections, and took approximately 20 minutes to complete. Section 1 recorded demographic data, perceived barriers to adopting evidence-based practice, and strategies used to overcome barriers. Section 2 required participants to rank (from one to five) how frequently they relied on different sources of information when making treatment decisions. Section 3 evaluated attitudes, knowledge and skill with regard to evidence-based practice, by asking each participant whether they '*agreed*', '*disagreed*' or were '*unsure*' about specific statements. Examples of statements used were: '*An electronic database such as PubMed can only be accessed from hospital and university libraries*' and '*The p value is a measure of reliability*'. These questions objectively tested participants' knowledge, and had correct/incorrect answers. Other questions asked about self-reported skills, ability and knowledge. For example, '*I am aware of and have used a range of electronic databases*' and '*I feel confident that I can critically appraise research evidence'*.

#### The adapted Fresno test

An adapted version of the Fresno test of competence in evidence-based medicine [23] was used to objectively measures skills and knowledge. The original Fresno test was designed to evaluate the effectiveness of a university curriculum on evidence-based medicine, and includes 12 short-answer questions, focussed around clinical scenarios relevant to family practice residents. Respondents are asked to write a focussed clinical question based on a scenario, list sources of information that could answer the question (for example, books and electronic databases), then comment on study designs and statistics reported in published papers. Internal consistency for the original Fresno test items, using Cronbach's alpha is 0.88, indicating a satisfactory level of agreement. Inter rater reliability is good to excellent, with correlations ranging from 0.72 to 0.96 for individual test questions, and 0.97 for total test scores [23].

For the current study, the original Fresno Test was adapted by AM to include clinical scenarios relevant to occupational therapists. Five of the 12 more advanced statistical questions were removed (for example, those about sensitivity, specificity, numbers needed to treat), since these would not be taught in the workshop curriculum. The new test, referred to hereafter as the 'Adapted Fresno Test', asked participants to choose one of two new scenarios and answer seven related questions (see Table 1). The Adapted Fresno Test took about 20 minutes to complete.

**Table 1:** Seven questions and clinical scenario from the Adapted Fresno Test

	**Question**
Q1	Write a focused clinical question for ONE scenario to help you organise a search of the literature.
	Sample scenario: *You have received a referral for a 38-year-old male client with chronic low back pain. He sustained his injury at work and is employed as a plumber. You are trying to decide if this man would benefit from using a TENS machine, in addition to attending a series of group education sessions on chronic pain management*.
Q2	Where might you find answers to these and other similar clinical questions? Name as many possible sources of information as you can, not just the ones you thinks are good sources. Describe the most important advantages and disadvantages of each source listed.
Q3	What type of study (design) would best answer your clinical question and why?
Q4	If you were to search Medline for original research to answer your question, describe the search strategy you might use. Be as specific as you can about topics and search categories (fields) you would use. Explain your rationale. Describe how you might limit your search.
Q5	When you find a report of original research on this question or any others, what characteristics of the study will you consider to determine if it is relevant?
Q6	When you find a report of original research, what characteristics of the study will you consider to determine if its findings are valid?
Q7	When you find a report of original research, what characteristics of the study will you consider to determine magnitude and significance (clinical and statistical)?

*Note*. TENS = transcutaneous electrical nerve stimulation

Three sets of different clinical scenarios were written for each test administration (i.e baseline, post-workshop and follow-up), to avoid a practice effect. Diagnoses included in the clinical scenarios were low back pain, traumatic brain injury, occupational overuse syndrome, depression, osteoarthritis and carpal tunnel syndrome. Interventions included transcutaneous electrical nerve stimulation, group education programs, workplace exercises, cognitive behaviour therapy, exercise programs and splinting (see example in Table 1). Analysis of internal consistency for the three versions of the Adapted Fresno Test yielded a Cronbach's alpha score ranging from 0.72 to 0.84, indicating an acceptable level of consistency for the adapted instrument [26].

The seven questions in the Adapted Fresno Test were scored using standardised grading criteria, similar to those reported by Ramos and colleagues [23]. The minimum test score was zero, and the maximum 156 for the seven questions (marking criteria available upon request). Each completed test took about 20 minutes to score. Scoring of the Adapted Fresno Test was evaluated for interrater reliability, and involved two raters independently scoring 20 completed tests (10 from pre-workshop and 10 from post-workshop) after receiving a two-hour training session [26]. Interrater reliability results ranged from poor to excellent depending on the test question being scored. Intraclass correlation coefficients (ICCs) ranged from 0.20 to 0.96 for the pre-workshop test (0.88 for the total score), and 0.41 to 0.92 for the post-workshop test (0.87 for the total score). In the pre-workshop survey, Questions 1, 4 and 5 had an ICC below 0.80 (0.20, 0.23 and 0.53 respectively). In the post-workshop survey, Questions 1 and 3 had an ICC below 0.70 (0.41 and 0.57 respectively). Further refinement of the Adapted Fresno Test scoring system is therefore indicated; implications for study results will be addressed later under 'limitations'.

### Activity diary

Behaviour change was measured using a concurrent activity diary, designed by AM for use in the study, and provided in paper or electronic format. Participants were asked to record only those activities that related to evidence-based practice, such as searching, reading research-related articles, critical appraisal and teaching others about evidence-based practice.

The following information was recorded in columns in participants' diaries, then subsequently analysed: date and nature of activity; what prompted the activity; start and finish times; whether the activity was conducted alone or not; if and how practice changed as a result of engaging in the activity. To improve accuracy, participants were asked to complete the activity diary contemporaneously for three weeks before and eight months after the workshop. Diaries were returned by fax, email or post on a bi-monthly basis. A research assistant contacted participants if their bi-monthly activity diary had not been returned.

### Written assignment

Completion of the assignment or CAT at the end of 2002 reflected engagement in the first three steps of the process of evidence-based practice (writing a focussed question, searching for evidence, and critically appraising the evidence). This outcome was recorded as completed/not completed.

### Analysis

Descriptive statistics, including means and percentages, were used to compare outcomes.

For the Adapted Fresno Test (the primary outcome measure), and based on statistical advice, paired t-tests were used to evaluate change in objective knowledge. Differences in mean total scores and confidence intervals (95% CI's) were calculated. A power calculation could not conducted in advance because the Adapted Fresno Test had not previously been used as an outcome measure, and expected means and standard deviations were unknown. Rather than performing a post-hoc power analysis, we examined the width of the confidence intervals (95%) for the estimated effects (pre-workshop to post workshop difference). When confidence intervals are reported in this way, a post-hoc power analysis is redundant [27]. Improvements of 10% (15.6 points) in the mean total score post-workshop, and 15% (23.4 points) at follow-up were considered clinically/educationally important, compared to baseline.

Non-parametric statistics were used for all other measures. Repeated measures were computed for the three occasions, rather than comparing paired samples, to reduce the risk of type 1 errors. Friedman's tests were used to test the hypotheses that over time: (a) more participants would be able to correctly answer the knowledge test questions, (b) more participants would feel confident and able to use published research in their work, (c) fewer participants would report barriers to evidence-based practice, and (d) more participants would conduct searches, read and appraise research than had done so before.

For knowledge test questions, the responses 'agree/disagree/unsure' were recoded as 'correct/incorrect'. For questions related to self-reported skills, confidence and ability, the responses 'agree/disagree/unsure' were recoded as 'yes/no'.

Non-responders were not included in analyses, post-intervention or at follow-up.

## Results

### Study sample

Demographic data for the 114 participants are presented in Table 2. The majority held an undergraduate degree in occupational therapy, worked full-time as a clinician in a metropolitan city, and had been graduated for 10 years or more. One third held a postgraduate qualification. One third worked in a regional or rural area. Just over 50% of the sample worked in the private sector.

**Table 2:** Demographic characteristics of participants (n = 114)

**Characteristic**	**n (%)**
**Level of initial occupational therapy qualification**	
Diploma	15 (13%)
Degree	99 (87%)
**Time since graduation**	
< 5 years	29 (25%)
≥ 5 but < 10 years	19 (17%)
≥ 10 years	66 (58%)
**Postgraduate Qualification**	
Yes	42 (37%)
No	72 (63%)
**Enrolled in Postgraduate Study**	
Yes	14 (12%)
No	100 (88%)
**Employment Status**	
Full-time	82 (72%)
Part-time ^1^	31 (27%)
Not currently employed	1 (1%)
**Geographical work location **(n = 113)	
Sydney metropolitan area	72 (64%)
Regional and rural areas	41 (36%)
**Primary work role **(n = 113)	
Clinician	70 (62%)
Manager	14 (12%)
Consultant	22 (19%)
Other	7 (7%)
**Primary work sector **(n = 113) ^2^	
Public	55 (49%)
Private	58 (51%)

*Notes*. ^1 ^Part-time employment = 25 hours or less per week. ^2 ^Public sector included government-funded health, community and vocational rehabilitation services, and universities. Private sector included private practice, private hospital settings, private rehabilitation providers and charitable/non government organisations.

Immediately post-workshop, 106 questionnaires and Adapted Fresno Tests were returned for analysis (92.9%). At follow-up, 51 (44.7%) of the possible 114 questionnaires and Adapted Fresno Tests were returned. For the activity diaries, 79 (69.3%) were returned for analysis in the first 8-week time-period post-workshop. At follow-up, only 40 of the possible 114 diaries (35.1%) were returned.

### Changes in knowledge

#### Adapted Fresno test

At baseline, the mean total score for the Adapted Fresno Test was 57/156 (range 0 to 126). Only 19% of participants achieved the 50% 'pass mark' of 78/156. Post-workshop, the mean total test score was 78/156 (range 32 to 124), with 77% achieving the pass mark. At follow-up, the mean total score was 82/156 (range 36 to 155), with 61% achieving the pass mark (see Table 3).

**Table 3:** Adapted Fresno Test scores (means, SD) over time

**Question**	**Time 1 Pre-Workshop ** (n = 114)	**Time 2 Post-Workshop ** (n = 106)	**Time 3 Follow-up ** (n = 51)
Q1 (0 to 12)	7.6 (7.8)	9.9 (1.6)	8.7 (1.7)
Q2 (0 to 24)	14.9 (6.6)	15.9 (4.2)	17.6 (5.9)
Q3 (0 to 24)	5.4 (7.0)	12.3 (4.6)	12.6 (6.6)
Q4 (0 to 24)	9.4 (7.1)	14.6 (6.1)	13.9 (6.2)
Q5 (0 to 24)	7.6 (5.6)	5.2 (4.5)	6.8 (6.5)
Q6 (0 to 24)	9.7 (8.1)	11.5 (7.5)	14.8 (7.6)
Q7 (0 to 24)	2.3 (3.9)	9.5 (8.4)	8.0 (6.1)
*Total *(0 to 156)	57.1 (26.7)	78.3 (18.6)	82.2 (24.5)

There were statistically significant and educationally important differences in knowledge when pre-workshop and post-workshop total scores were compared (mean difference 20.6 points, 95% CI, 15.6 to 25.5), and when pre-workshop and follow-up total scores were compared (mean difference 23.1 points, 95% CI, 14.7 to 31.6, see Table 4). Although differences between post-workshop and follow-up were small and non-significant, the knowledge gains were maintained after eight months.

**Table 4:** Adapted Fresno Test scores, mean differences over time (paired *t*-tests)

	***Mean Diff***	***95% CI***	***t***	***DF***	***p***
*Pre- to Post-Workshop*					
Total score (0–156)	20.6	(15.6 – 25.5)	8.3	105	0.0001**
Appraisal sub-score^1 ^(0–77)	6.4	(3.0 – 9.8)	3.8	105	0.0001**
					
*Post-Workshop to Follow-Up*					
Total score (0–156)	1.2	(-6.0 – 8.5)	0.3	50	0.734
Appraisal sub-score^1 ^(0–77)	2.6	(-1.9 – 6.9)	1.2	50	0.250
					
*Pre-Workshop to Follow-Up*					
Total score (0–156)	23.1	(14.7 – 31.6)	5.5	50	0.0001**
Appraisal sub-score^1 ^(0–77)	9.9	(4.9 – 15.0)	3.9	50	0.0001**

*Notes*. ^1^Appraisal sub-score = questions 5, 6 and 7. ** Significant at 0.05

There was no important difference in the baseline scores of recent graduates compared to more experienced therapists. At baseline, the mean total score on the Adapted Fresno Test for recent graduates (previous five years) was 59.8, compared to 56.5 for all others.

The three questions testing critical appraisal skills and knowledge were analysed separately for the Adapted Fresno Test (Questions 5, 6 and 7 = sub-total of 72). There were statistically significant differences in appraisal knowledge when pre-workshop and post-workshop sub-totals were compared (mean difference 6.4 points, 95% CI, 3.0 to 9.8), and when pre-workshop and follow-up sub-totals were compared (mean difference 9.9 points, 95% CI, 4.9 to 15.0, see Table 4). Differences between post-workshop and follow-up were small and non-significant. The mean sub-total for these three questions did not, however, reach the 50% pass mark of 36/72 even at follow-up.

#### Other knowledge test questions

As hypothesised there were statistically significant differences in the proportion of therapists over time who could correctly answer questions about a good clinical question, PubMed and Medline, Cochrane reviews, confidence intervals, p-values and single case design research (see Table 5). Knowledge gains were maintained at follow-up.

**Table 5:** Differences over time in proportions answering knowledge questions correctly (n, %), Friedman's Test

**Questions** **(abbreviated)**	**Time 1 Pre** (n = 114)	**Time 2 Post** (n = 106)	*% change*	**Time 3** **Follow-up** (n = 51)	*% change*	**DF**	**χ^2^**	**p**
Q1. *A good clinical question consists of an intervention, target population, outcome, and comparison intervention (Agree)*								
**Correct**	53 (46.9)*	**105 (99.1)**	**+55**	**48 (94.1)**	**+5**	2	44.9	0.0001**
Incorrect	60 (53.1)*	1 (0.9)		3 (5.9)				
Q2. *Databases of primary medical literature, such as Medline/Pubmed generally contain a compilation of only high quality evidence (Disagree)*								
**Correct**	**8 (7.0)**	**42 (39.6)**	**+7**	**35 (70.0)^2^**	**+30**	2	21.8	0.0001**
Incorrect	106 (93.0)	64 (60.4)		15 (30.0)^2^				
Q3. *An electronic database such as PubMed can only be accessed from hospital and university libraries (Disagree)*								
**Correct**	**26 (22.8)**	**102 (96.2)**	**+73**	**47 (92.2)**	**-4**	2	68.7	0.0001**
Incorrect	88 (77.2)	4 (3.8)		4 (7.8)				
Q4. *The Cochrane Database is a good place to look for reviews of high quality research based on clearly stated criteria, in many areas of clinical practice (Agree)*								
**Correct**	**46 (40.4)**	**98 (92.5)**	**+53**	**45 (88.2)**	**-5**	2	40.5	0.0001**
Incorrect	68 (59.6)	8 (7.5)		6 (11.8)				
Q5. *Confidence intervals are a measure of clinical significance, and provide a way of estimating where the 'true' result for any population lies (Agree)*								
**Correct**	**20 (17.5)**	**92 (86.8)**	**+69**	**41 (80.4)**	**-7**	2	63.5	0.0001**
Incorrect	94 (82.5)	14 (13.2)		10 (19.6)				
Q6. *The p-value is a measure of reliability (Disagree)*								
**Correct**	**10 (8.8)^1^**	**61 (57.5)**	**+49**	**20 (40.0)**	**-18**	2	33.3	0.0001**
Incorrect	103 (91.2)^1^	45 (42.5)		30 (60.0)				
Q7. *Single case designs are regarded as a similar level of evidence to randomised controlled trials (Disagree)*								
**Correct**	**72 (63.7)^1^**	**101 (95.3)**	**+31**	**50 (98.0)**	**+3**	2	22.5	0.0001**
Incorrect	41 (36.3)^1^	5 (4.7)		1 (2.0)				

*Note*: Responses recoded from 'Agree/Disagree/Unsure' to 'Correct/Incorrect'. ^1^n = 113. ^2^n = 50. **Significant at 0.05

### Self-reported skills, knowledge and confidence

Based on the self-report questionnaire, there was an immediate increase in the proportion of therapists who felt their skills, knowledge and confidence had improved post-workshop (see Table 6). These changes and proportions were maintained at follow-up. As hypothesised, there were statistically significant differences in the proportion of therapists over time who felt confident: generating a clinical question, using electronic databases, conducting databases searches alone, using a computer and the internet, and appraising research.

**Table 6:** Differences over time in proportions reporting improved confidence and abilities (n, %), Friedman's Test

**Questions**	**Time 1 Pre** (n = 114)	**Time 2 Post** (n = 116)	*% change*	**Time 3 Follow up** (n = 51)	*% change*	**DF**	**χ^2^**	**P**
*Q1. I feel confident that I can generate a clinical question*								
**Yes**	**38 (33.3)**	**87 (82.1)**	**+48.8**	**48 (94.1)**	**+12.0**	2	51.2	0.0001**
No	76 (66.7)	19 (17.9)		3 (5.9)				
*Q2. I am aware of and have used a range of electronic databases*								
**Yes**	**51 (44.7)**	**99 (93.4)**	**+48.7**	**51 (100.0)**	**+6.6**	2	52.3	0.0001**
No	63 (55.3)	7 (6.6)		0 (0.0)				
*Q3. I am able to conduct a computer search on my own*								
**Yes**	**49 (43.0)**	**90 (84.9)**	**+42.0**	**47 (92.2)**	**+7.3**	2	38.0	0.0001**
No	65 (57.0)	16 (15.1)		4 (7.8)				
*Q4. I am confident about my general computer skills such as using the internet*								
**Yes**	**86 (75.4)**	**85 (80.2)**	**+4.8**	**49 (96.1)**	**+15.9**	2	9.1	0.010**
No	28 (24.6)	21 (19.8)		2 (3.9)				
*Q5. I feel confident that I can critically appraise research evidence*								
**Yes**	**20 (17.5)**	**25 (23.6)**	**+6.1**	**18 (36.0)^1^**	**+11.7**	2	13.5	0.0001**
No	94 (82.5)	81 (76.4)		32 (64.0) ^1^				

*Note*: Responses recoded from Agree/Disagree/Unsure to Yes/No. ^1^n = 50. ** Significant at 0.05

Critical appraisal was challenging for many participants. Less than one quarter of participants felt confident with their appraisal skills pre-workshop (18%) and post-workshop (24%). However, the proportion rose a little at follow-up to 36%. These differences over time were statistically significant (see Table 6)

### Change in attitude and barriers reported

Immediately after the workshop, a higher percentage of participants (94%) reported lack of time as a barrier than had done so before (75%), as they became aware of what evidence-based practice involved. Lack of time remained an ongoing concern for 88% at follow-up. These differences were statistically significant (see Table 7).

**Table 7:** Differences over time in proportions reporting barriers to evidence-based practice (n, %), Friedman's Test

**Key barriers**	**Time 1 Pre-Workshop ** (n = 114)	**Time 2 Post-Workshop ** (n = 106)	**Time 3 Follow-up ** (n = 51)	**DF**	**χ^2^**	**P value**
Lack of time #	86 (75%)	100 (94%)	45 (88%)	2	12.8	0.002**
Large workload/caseload	76 (67%)	79 (75%)	31 (61%)	2	1.1	0.568
Limited critical appraisal skills	68 (60%)	69 (65%)	21 (41%)	2	9.2	0.010**
Limited searching skills	69 (61%)	56 (53%)	12 (24%)	2	20.9	0.0001**
Difficulty accessing journals	51 (45%)	45 (43%)	18 (35%)	2	2.7	0.259

*Notes*. # Participants were asked to '*list any barriers to implementing or adopting evidence-based practice, which apply to your workplace*" and to choose as many items as they wished from a 17-item list. Therefore, numbers do not add up to 100%. **Significant at 0.05.

As hypothesised, there was a significant difference (a decrease) in the proportion of therapists who felt their searching and appraisal skills were a barrier to evidence-based practice (see Table 7).

Searching was perceived to be less of a problem than appraisal. The proportions reporting limited search skills as a perceived barrier changed from 61% pre-workshop, to 53% post-workshop, and 24% at follow-up. The proportions reporting limited appraisal skills as a perceived barrier changed from 60% pre-workshop, to 65% post-workshop, and 41% at follow-up.

After eight months, there was also a non-significant decrease in the proportion of participants that reported difficulty accessing journals (see Table 7).

### Change in behaviour and activity levels

Table 8 summarises the frequency of searching, reading, and critical appraisal activities, and time spent teaching others about evidence-based practice. Only a small proportion of participants used the skills and knowledge acquired at the 2-day workshop although 23 participants (20.2%) completed their assignment.

**Table 8:** Nature and frequency of participant behaviour (activity levels) over time (n, %), Friedman's Test

	**Time Periods**							
	
**Behaviour and Frequency**	**Time 1 Pre** *3 weeks* (n = 102)	**Time 2 Mar–Apr ** *8 weeks* (n = 79)	(Time 3)* May–Jun *8 weeks* (n = 57)	(Time 4)* Jul–Aug *8 weeks* (n = 47)	**Time 5 Sep–Oct ** *8 weeks* (n = 40)	**DF**	**χ^2^**	**p**
**Searching**								
Nil occasions	46 (45.1%)	25 (31.6%)	28 (49.1%)	21 (44.6%)	25 (62.5%)			
Once	28 (27.4%)	22 (27.8%)	16 (28.1%)	14 (29.8%)	4 (10.0%)	2	9.4	0.009**
Twice or more	28 (27.4%)	32 (40.5%)	13 (22.8%)	12 (25.5%)	11 (27.5%)			
**Reading**								
Nil occasions	71 (69.6%)	58 (73.4%)	39 (68.4%)	39 (83.0%)	24 (60.0%)			
Once	16 (15.6%)	14 (17.7%)	10 (17.5%)	5 (10.6%)	7 (17.5%)	2	3.5	0.172
Twice or more	15 (14.7%)	7 (8.9%)	8 (14.0%)	3 (6.3%)	9 (22.5%)			
**Appraisal**								
Nil occasions	96 (94.1%)	65 (82.3%)	46 (80.7%)	42 (89.4%)	33 (82.5%)			
Once	3 (2.9%)	8 (10.1%)	5 (8.8%)	3 (6.4%)	6 (15.0%)	2	4.5	0.105
Twice or more	3 (2.9%)	6 (7.6%)	6 (10.5%)	2 (4.3%)	1 (2.5%)			
**Teaching others**								
Nil occasions	97 (95.1%)	65 (82.3%)	46 (80.7%)	45 (95.7%)	39 (97.5%)			
Once	4 (3.9%)	6 (7.6%)	8 (14.0%)	2 (4.2%)	0 (0.0%)	2	3.4	0.179
Twice or more	1 (1.0%)	8 (10.1%)	3 (5.3%)	0 (0.0%)	1 (2.5%)			

*Notes*. *Searching *refers to use of online databases and websites, in order to answer a clinical question. *Reading *refers to the reading of research-related textbooks, articles and other documents without using a critical appraisal checklist. *Critical appraisal *refers to reading and interpreting a research article, while simultaneously using a critical appraisal checklist. *Teaching *refers to any teaching, or preparation for teaching, related to evidence-based practice. * (Time 3 and Time 4 not included in repeated measures analysis, only Time 1, Time 2 and Time 5). ** Significant at 0.05

The only statistically significant difference was a decrease – not an increase as hypothesised – in the proportion of therapists engaged in searching between Time 1 and Time 5. Nonetheless, searching was the most popular activity followed by reading without appraisal. Between 23% and 41% of participants searched electronic databases twice or more over eight weeks, and between 10% and 30% searched at least once over eight weeks. However, for critical appraisal, only 3% to 11% of participants engaged in this activity at least once over eight weeks. The majority, between 83% and 89%, did not participate in any critical appraisal in the eight-week time periods. Research utilisation behaviours were low initially and remained low.

## Discussion

The aim of this study was to evaluate the effect of a 2-day interactive workshop plus follow-up support – a 'multi-faceted' intervention – on the knowledge and skills, attitudes and behaviour of occupational therapists.

There were several key findings. First, improvements in knowledge were statistically and educationally significant, and these changes were maintained at follow-up. Second, attitudes to, and confidence with searching and appraisal improved over time, with more participants feeling confident searching for, than appraising evidence at all stages of the study. Third, the frequency of evidence-seeking behaviour and appraisal changed little over eight months.

### Gains in knowledge were statistically significant and educationally important

Based on the Adapted Fresno Test, there were significant changes in knowledge over time. Furthermore, changes in the Adapted Fresno Test total score were consistent with other measures of knowledge: more therapists correctly answered the knowledge test questions and reported better knowledge and skills over time.

Nonetheless, it is possible that a learning effect occurred with each of these measures and contributed to the positive findings. Therefore, the findings should be interpreted with caution. In addition, some of the change reported for the Adapted Fresno Test may reflect measurement error, because of less-than-perfect interrater reliability for four of the seven questions. Scoring guidelines for Questions 1, 3, 4 and 5 require further refinement before the test can be published and used by researchers.

### Critical appraisal: More challenging to learn than searching?

The results indicate that participants found appraisal more challenging to learn than searching. A large proportion (65%) still felt their appraisal skills were a barrier to evidence-based practice even after the 2-day workshop. However, participants learned about different research methods, both qualitative and quantitative, as well as how to interprete p-values and confidence intervals in a relatively short time. Most recognised that they would have to practice these skills back at work, if they wished to confidently appraise research on their own. They also realised how much time this additional learning would take out of their already busy schedule.

Unfortunately, activity diaries show that few participants found time to read or appraise research, in the eight months that followed. Over 60% did not read, and over 80% did not appraise any research literature. Additional studies, both quantitative and qualitative, are needed to determine the most effective way of establishing reading and appraisal habits. None of the current study participants managed to establish a regular journal club in their workplace over eight months. A recent study of physiotherapy departments [28] found that journal clubs existed in 42% of responding departments in England, but only 19% of those surveyed in Australia. However, relatively few departments used an evidence-based format involving appraisal. Currently, there are descriptive examples of multidisciplinary [29] and cross-regional [30] journal clubs in the allied health and nursing literature, but no evaluations of outcome. Without regular events like a journal club, allied health professionals will potentially lose these skills, and individuals will be less likely to move on and implement evidence in practice.

### Behaviour and activity levels changed little

Disappointingly, our findings about activity levels concur with those of Forsetlund and colleagues [16], who targetted public health physicians in Norway. In their randomised controlled trial, education was followed by extensive support including newsletters, an electronic discussion list, assignments and free access to several databases. Despite the lengthy follow-up period (1.5 years), there was little difference in the frequency of searching or the application of evidence over time, or between intervention and control groups. Knowledge improved, but not the use of evidence.

Forsetlund and colleagues proposed that despite their negative findings, changes in knowledge and attitude may still be important pre-requisites for evidence-seeking behaviour, like a developmental stage. Further, they proposed that 1.5 years after learning essential knowledge and skills may still be too early to observe evidence being used in practice.

The findings of our study support the views of Forsetlund and colleagues. Developing skills for evidence-based practice involves a major change in values and priorities, habits and routines at an individual and organisational level. In the current study, it was not anticipated that occupational therapists would apply evidence in daily practice with patients after eight months, only that they would look for, appraise and summarise best evidence. Using Prochaska and DiClemente's staged model of change [22], most participants in the current study moved from the stage of contemplation to the stage of action. In real terms, they moved from thinking about, and taking an interest in evidence-based practice, to attending a workshop, planning and then (for some at least), working on a CAAT. However, study participants had to overcome barriers such as lack of time and large workloads, and make time to apply their skills and knowledge back at work. While more than 50% of the sample engaged in searching post-workshop, less than 20% reached the stage of critical appraisal, and only 20% completed their CAT within the eight months.

### Limitations of the study

As with all research, this study had limitations. First, no control group was used for comparison. Therefore, we do not know if changes in knowledge would have occurred anyway, without the intervention. However, that seems unlikely based on other studies that used a control group [6,16] and reported similar knowledge outcomes.

Second, all participants were self-selected occupational therapists from one state in Australia. Randomly selected participants may have been less motivated to learn, and the results less positive. However, care was taken during recruitment to minimise recruitment bias and obtain a representative sample. Table 2 indicates that the sample was demographically representative of occupational therapists across NSW with over two- thirds working full-time in the Sydney metropolitan area [31]. Equal proportions of participants worked in the public and private sector, also consistent with OT Australia NSW membership statistics at the time of data collection. Nonetheless, occupational therapists in the study chose to participate and the self-selected sample from one state in Australia needs to be considered when interpreting results.

A third limitation relates to the primary outcome measure. While the interrater reliability of the Adapted Fresno Test total score is acceptable (0.87 to 0.88), further work is required to improve the scoring system and reliability of four sub-test questions. For this reason, only mean differences and confidence intervals for the total score have been reported.

A fourth limitation is the possibility of a learning effect from repeated administration of the outcome measures. Repeated administration of measures, particularly those focussing on knowledge, may have over-estimated the treatment effect.

Finally, the follow-up rate in the current study was low. Only 51 participants (44.7%) returned their survey and Adapted Fresno Test for analysis at follow-up, introducing another methodological bias. Those who did not return their documentation are likely to have been less engaged, and possibly less knowledgeable and confident than those who responded. This loss to follow-up is also likely to over-estimate the effect of the intervention and needs consideration when interpreting results.

## Conclusion

The focus of this study was on change – in knowledge, skills, attitudes and behaviour. The intervention helped occupational therapists to improve their knowledge, skills and confidence in relation to the first three steps of evidence-based practice. Furthermore, knowledge and skills were retained over eight months.

The study provides practical strategies for measuring change in skills and knowledge, and the frequency of evidence-seeking behaviour. Some of these measures can be used to evaluate the effectiveness of undergraduate and graduate education programs.

Disappointingly, the intervention had little impact on behaviour over eight months. Most participants were unable to establish a regular pattern of searching, reading and appraisal even when their behaviour was monitored.

The ongoing challenge for educators, researchers and managers is how to help clinicians establish new routines and priorities around evidence-based practice. The reality is that behaviour change of this nature probably takes years, not months. Allied health educators and learners may find it helpful to examine outcomes from the current study, discuss the process and stages of behaviour change, and plan realistic longer term goals.

## Competing interests

The author(s) declare that they have no competing interests.

## Authors' contributions

Annie McCluskey designed, conducted, analysed and wrote up the results of this study. Meryl Lovarini developed teaching materials for the workshop, provided support to participants during the study, assisted with data collection, analysis and writing up.

## Pre-publication history

The pre-publication history for this paper can be accessed here:


